# Erosion of universal health coverage and trend in the frequency of physician consultations in Spain

**DOI:** 10.1186/s12939-020-01234-z

**Published:** 2020-07-13

**Authors:** Lourdes Lostao, Elena Ronda, Cruz Pascual, Lucía Cea-Soriano, Almudena Moreno, Enrique Regidor

**Affiliations:** 1 Department of Sociology, Medical Sociology, Universidad Pública de Navarra, Pamplona, Spain; 2 I-COMMUNITAS-Institute for Advanced Social Research, Universidad Pública de Navarra, Pamplona, Spain; 3 Department of Preventive Medicine and Public Health, Universidad de Alicante, Alicante, Spain; 4 CIBER Epidemiología y Salud Pública (CIBERESP), Madrid, Spain; 5 Department of Preventive Medicine and Public Health, Universidad Complutense de Madrid, Madrid, Spain; 6 Instituto de Investigación Sanitaria del Hospital Clínico San Carlos (IdISSC), Madrid, Spain

## Abstract

**Background:**

We studied the frequency of physician visits in the native and immigrant populations in Spain before and after implementation of a governmental measure to restrict the use of public healthcare services by undocumented immigrants beginning in 2012.

**Methods:**

Data were taken from the 2009 and 2014 European Health Surveys carried out in Spain. We investigated any physician consultation in the last 4 weeks before the interview, as well as visits to a family physician, public specialist physician and private specialist physician. We estimated the frequency of visits in 2009 and in 2014 in the native and immigrant populations and the difference in the frequency between the two populations, by calculating the percentage ratio estimated by binomial regression and adjusted for different confounders that are indicators of the need for assistance.

**Results:**

The percentage of persons who consulted any physician in 2009 and 2014 was 31.7 and 32.9% in the native population, and 25.6 and 30.1% in the immigrant population, respectively. In the immigrant population, the frequency of visits to the general practitioner and public specialist physician increased, whereas in the native population only public specialist physician visits increased. The frequency of private specialist visits remained stable in both populations. After adjusting for the indicators of need for healthcare, no significant differences between the immigrant and native populations were seen in the frequency of visits, except for private specialist consultations, which were less frequent among immigrants.

**Conclusion:**

The restriction of universal healthcare coverage in Spain did not reduce the frequency of physician visits between 2009 and 2014, as the frequency of these consultations was seen to increase in both the native and immigrant populations.

## Introduction

The global financial crisis of 2008 led to many changes in the social policies of most high-income countries. In the case of Spain, there was a major reduction of the public budget for social services, education and health [1–3]. For example, mean annual growth of public expenditure on health per inhabitant dropped from 11.2% between 2005 and 2008 to − 2.2% between 2009 and 2012 [4]. This reduction in resources assigned to the healthcare system could have led to reduced population access to health services.

Furthermore, the reduced budget could have had a greater effect in population groups with more adverse socioeconomic conditions, on whom the crisis has had a larger impact. Some researchers have asserted that people who have lost their jobs prefer to use their time looking for employment rather than seeking medical care [5]. However, two studies performed using information from the National Health Surveys of 2006 and 2012 showed a slight reduction in the frequency of physician visits in 2012 with respect to 2006, but an absence of significant changes in the pattern of use of services, both according to social class and native/immigrant status [6, 7].

These investigations could not evaluate the impact of other measures the Spanish government implemented in 2012 [8]. Specifically, a measure that restricted the use of public healthcare services by all immigrants who did not belong to the Social Security system because they were unemployed and for undocumented immigrants. That is, beginning in 2012 public healthcare ceased to be a universal right in Spain, since unemployed immigrants undocumented immigrants and were stripped of that right.

Until 2012, the coverage of the system was universal. All individuals living in Spain were entitled by law to receiving free access to the system irrespective of personal wealth, labor status or administrative. In the case of undocumented immigrants they were entitled to the same bundle of services as Spanish natives with the only requirement of being registered as residents in the city hall where they resided. In 2002, this situation for undocumented immigrants changed. The Spanish government implemented a reform of the health system with the approval of Royal Decree 16/2012. The new law, which came into effect in September 12,012, restricted free access to the health care services for the population of undocumented immigrants. Also unemployed immigrants were excluded from the right to health care when their unemployment benefit ran out. With the new law, individuals losing entitlement to comprehensive care retain protection if they are younger than 18 years; during pregnancy, delivery, and post-partum period; and for emergency care after serious illness or injury.

The government justified this measured by the need to reduce public spending due to the great impact of the 2008 financial crisis in Spain, although the government did not present estimates of the amount that was supposed to save public spending with this measure. According to a report from the Council of Europe, although the right to health is guaranteed by international and European human rights instruments, Royal Decree 16/2012 established an important restriction of access to the health system to undocumented migrants [9]. Within a human rights framework, some authors have point out the contradiction between the standards established in international and European strategic documents and the legislative framework related to access to health care present in various European countries [10]. Those authors mentioned that the Spanish Public Health Care System, previous to the RDL 16/2012, belonged to the group of countries that allowed a relatively high level of access to health care for this population group. However, the changes introduced in the RDL 16/2012 modify substantially this position towards the category of countries with the highest level of restriction regarding access to health care [2].

Given that there are no population sources in which it is possible to identify the administrative status of immigrants, an alternative is to study the entire immigrant population. However, there are no studies on the effect that this measure has had on the frequency of medical consultations in the immigrant population in relation to the native population. The objective of this study was to estimate the frequency of physician visits in the native and immigrant populations in Spain before and after implementation of a governmental measure to restrict the use of public healthcare services by undocumented immigrants beginning in 2012.

## Methods

The data were taken from the 2009 and 2014 European Health Surveys in Spain carried out by the National Statistics Institute. Both surveys were carried out with the same methodology and therefore allow the comparison of the results. The sampling framework was made up of the Spanish non-institutionalized population aged 16 or over. The surveys were performed using a two-stage sample design. The first-stage units were the census sections and the second-stage units were the households in each of the selected sections. The households were selected by simple random sampling, and one adult aged 16 or over was selected within each household. Information was collected by face-to-face interviews. The selected sample of each year is representative of the national population. The response rate was 73.3% in 2009 and 74.6% in 2014. The interviews were 22,188 in 2009 and 22,842 in 2014. A detailed account of those surveys and its data structure can be found in the website of National Statistics Institute [11]. For the present study, we selected subjects under age 75 since the probability of being institutionalised increases after that age. Therefore, we exclude 10% of those interviewed in each year as they are 75 years of age or older.

The health survey respondents were interviewed about the frequency of their medical visits. Those who had any medical visit in the last 4 weeks were asked if the physician consulted at the most recent visit was a family doctor or a specialist. They were then asked if that physician was in the public health system, was from a private health insurance company, or if they had paid directly for the consultation. In the first case, the physician visit was considered to be publicly financed, while in the latter two cases the visit was considered to be privately financed. Family doctors only carry out their professional work in the public system, so all visits to a family doctor were considered publicly financed. We studied the following four dependent variables referring to medical consultations made in the last 4 weeks: visit to any physician, visit to a family physician, visit to a specialist physician in the public system, and visit to a specialist in the private system. The consultation with the dentist, stomatology, and dental hygienist are included within the visits to private medical specialists, since these consultations are not funded by the Spanish Public Health Care System.

The interviewees were grouped into two categories according to birth: natives, if they were born in Spain, and immigrants if they were born outside of Spain. Also included were sex, age and educational level of respondents, as well as household income, as possible confounding variables in the relationship between place of birth and physician visit. Respondents were grouped into four educational categories based on the highest level completed. In the European Health Surveys in Spain, household income was not obtained by an open question, but rather respondents had to select an income category from the different intervals shown on the questionnaire. For the statistical analysis, subjects were grouped into four categories. Given the importance of household size when assessing the influence of income, number of household members was included as a confounding variable.

The variables indicating need for care were self-reported health and the presence of any long-term health problem. Self-perceived health was measured by the following question: “Over the last 12 months, would you say your health has on the whole been very good, good, fair, poor or very poor?” Respondents had to choose one of these five alternatives. The presence of a long-term health problem was obtained by the following question: Do you have any chronic or long-term disease or health problem? (“long term” was understood to refer to a disease or health problem that had lasted or was expected to last 6 months or longer). Respondents replied yes or no.

### Statistical analysis

For each year, we obtained the distribution of the native and immigrant populations according to the study variables. Possible differences between the two distributions were compared using the chi square of heterogeneity. We then estimated the frequency of visits with each type of physician in 2009 and 2014 and measured if there was a statistical difference between the 2 years using the chi square. We then obtained the magnitude of the relationship between each of the independent variables of adjustment and/or indicators of need for care and the frequency of consultations with any physician. For this purpose we calculated the percentage ratio adjusted for age estimated by binomial regression. Finally, we estimated the magnitude of the relationship between place of birth (independent variable) and frequency of consultation with each type of physician (dependent variables) by calculating the percentage ratio estimated by binomial regression, taking the native population as the reference group. In the first model we included age and sex as the cofounder variables. In the second model we added other confounder variables: educational level, income and household size, self-reported health and the presence of any long-term disease. We have chosen the calculation of the percentage ratio by binomial regression instead of the odds ratio by logistic regression. The reason for this is that the odds ratio overestimates the magnitude of the relationship when the value of the dependent variable is greater than 10% [12, 13]. And this happens in our study with the frequency of some of the types of consultation with physician analyzed. Sampling weights were used in all statistical analyzes. The National Statistics Institute developed the sampling weights incorporated the design factor using calibration techniques using CALMAR software [14].

## Results

Table 1 shows the size and distribution of the native and immigrant populations according to the different study variables in the two periods. In both 2009 and 2014, the immigrant population was younger, had a larger percentage of men, lower percentage of subjects with high income, lower percentage of those with tertiary education, and lower percentage of subjects with some chronic disease. In 2009, the immigrant population had a smaller percentage of subjects with negative self-reported health, but in 2014 this variable was not significantly different between the two populations.

**Table 1:** Distribution of the native-born and immigrant population by different categories of the variables of analysis. Spain, 2009 and 2014

Variables	2009			2014		
	Natives	Immigrants	*p* value*	Natives	Immigrants	*p* value*
Number of subjects	16,913	3104		17,610	2907	
**Age (years)**			< 0.001			< 0.001
16–24	12.2	16.4		12.3	15.4	
25–34	19.1	34.4		15.8	25.3	
35–44	21.1	26.6		21.3	28.5	
45–54	19.5	12.9		20.4	18.3	
55–64	15.8	6.1		16.7	8.3	
65–74	12.3	3.6		13.5	4.2	
**Sex**			0.0198			< 0.001
Women	50.4	48.1		50.5	45.6	
Men	49.6	51.9		49.5	54.4	
**Income**			< 0.001			< 0.001
High	22.3	13.6		23.5	9.9	
Medium-high	19.1	18.3		22.1	18.4	
Medium-low	19.4	20.2		17.8	20.7	
Low	20.6	30.4		16.3	29.5	
Missing	18.6	17.5		20.3	21.5	
**Educational level**			< 0.001			< 0.001
Tertiary	25.0	23.3		20.7	16.7	
Upper secondary	22.2	33.9		29.4	36.0	
Lower secondary	19.4	21.1		25.4	23.9	
Elementary	33.4	21.7		24.5	23.4	
**Self-reported health**			< 0.001			0.1554
Fair/poor/very poor	22.6	16.7		25.1	23.9	
Very good/good	77.4	83.3		74.9	76.1	
**Any long-term health problem**			< 0.001			< 0.001
Yes	50.2	32.2		57.6	47.4	
No	49.8	67.8		42.4	52.6	

*p value of the chi square of heterogeneity

Table 2 shows the frequency of physician consultations in the 4 weeks before the interview in 2009 and 2014 in the native and immigrant populations. The percentage of people who made any medical consultation was higher in 2014 than in 2009, especially in the immigrant population. In the native population, the frequency of family physician consultations was similar in both periods, whereas consultations with a public specialist physician were more frequent in 2014 than in 2009. Specifically, the percentage of persons who had consulted a family physician, public specialist or private specialist in 2009 was 26.7, 8.9 and 3.1%, while the respective values for 2014 were 26.6, 11.3 and 2.8%. In the immigrant population, consultations with family physicians and with public specialist physicians were more frequent in 2014 than in 2009. Specifically, the percentage of persons who had consulted a family physician, public specialist physician and private specialist in 2009 was 21.0, 8.3% and 1.2%, respectively, while the corresponding values for 2014 were 25.6, 10.0 and 1.3%. The difference between the percentages in 2014 and 2009 was statistically significant, except for consultation with the physician in the native population and with the private specialist in both populations.

**Table 2:** Frequency (in percentage) of physician consultations in the native-born population and in immigrants, and *p* value of percentage differences. Spain, 2009 and 2014

Type of consultation	Percentage		*P* value for difference
	2009	2014	
**Any consultation**			
Native	31.7	32.9	< 0.025
Immigrant	25.6	30.1	< 0.001
**Consultation with family physician**			
Native	26.7	26.6	0.970
Immigrant	21.0	25.6	< 0.001
**Consultation with public specialist physician**			
Native	8.9	11.3	< 0.001
Immigrant	8.3	10.0	< 0.025
**Consultation with private specialist physician**			
Native	3.1	2.8	0.100
Immigrant	1.2	1.3	0.750

The percentage ratio, which evaluates the relationship of age, sex, educational level, household income, self-reported health and presence of any chronic disease to consultation with a physician in the 4 weeks before the interview, is shown in Table 3. The percentage ratio increases with age and was significantly higher in women (1.45 in 2009 and 1.37 in 2014 with respect to men), in subjects with lowest educational level (1.12 in 2009 and 2014 with respect to those with tertiary education), in subjects with lowest income (1.15 in 2009 and 1.34 in 2014 with respect to those with in the highest income interval), in those with negative self-reported health (2.08 in 2009 and 2.09 in 2014 with respect to those with positive self-report), and in those with any long-term disease or health problem (1.99 in 2009 and 2.07 in 2014 with respect to those without a long-term condition).

**Table 3:** Relationship of different variables to consultation with any physician in the 4 weeks before the interview. Percentage ratio (PR) y 95% confidence interval (95% CI)

	2009		2014		
	PR^a^	95% CI	PR^a^		95% CI
**Age (years)**					
16–24	1.00		1.00		
25–34	1.14	1.04–1.25	1.11	1.02	1.21
35–44	1.24	1.21–1.28	1.18	1.08	1.28
45–54	1.48	1.35–1.62	1.36	1.26	1.48
55–64	1.85	1.70–2.02	1.69	1.55	1.83
65–74	2.40	2.21–2.62	2.06	1.90	2.23
**Sex**					
Men	1.00		1.00		
Women	1.45	1.39–1.52	1.37	1.32	1.42
**Self-reported health**					
Very good/good	1.00		1.00		
Fair/poor/very poor	2.08	1.99–2.18	2.09	2.01	2.18
**Any long-term health problem**					
No	1.00		1.00		
Yes	1.99	1.90–2.09	2.07	1.97	2.17
**Income**					
High	1.00		1.00		
Medium-high	1.03	0.96–1.10	1.10	1.03	1.17
Medium-low	1.10	1.03–1.17	1.19	1.12	1.27
Low	1.15	1.08–1.23	1.34	1.26	1.43
Missing	0.98	0.91–1.05	1.02	0.95	1.08
**Educational level**					
Tertiary	1.00		1.00		
Upper secondary	1.05	0.98–1.12	1.01	0.95	1.07
Lower secondary	1.08	1.01–1.16	1.11	1.04	1.18
Elementary	1.12	1.06–1.19	1.12	1.06	1.19
Missing	1.05	0.52–2.11	..	..	..

^a^The percentage ratio has been estimated with a regression model for each of the independent variables that appear in the table, with the dependent variable being any consultation with any physician. In the regression model with age, the independent variable is only age, but in models with the other independent variables, age has also been included as an adjustment variable

The relationship of immigrant vs. native status to frequency of physician consultations in 2009 and 2014 can be seen in Table 4. After adjusting for all the variables, no significant differences were seen in the frequency of consulting any physician. The percentage ratio was 0.95 [95% confidence interval (95%CI) 0.79–1.15] in 2009 and 0.96 (95%CI 0.91–1.02) in 2014. Likewise, after full adjustment, no significant differences were observed in the frequency of consultation with a family physician, [percentage ratio 0.99 (95%CI 0.92–1.06) in 2009 and 1.02 (95%CI 0.96–1.09) in 2014], or with a public specialist physician [percentage ratio was 1.06 (95%CI 0.94–1.21) in 2009 and 0.92 (95%CI 0.82–1.04) in 2014]. However, the frequency of consultation with a private specialist was significantly lower in immigrants than in the native-born population in the fully adjusted model [percentage ratio 0.38 (95%CI 0.27–0.53) in 2009 and 0.51 (95%CI 0.36–0.71) in 2014.

**Table 4:** Relationship of place of birth to different types of physician consultation in the 4 weeks before the interview. Percentage ratio (PR) y 95% confidence interval (95% CI)

	2009				2014			
	Model 1		Model 2		Model 1		Model 2	
	PR^a^	95% CI	PR^a^	95% CI	PR^**a**^	95% CI	PR^a^	95% CI
**Any consultation**								
Native	1.00		1.00		1.00	**–**	1.00	
Immigrant	0.90	0.85–0.96	0.95	0.79–1.15	1.00	0.94–1.06	0.96	0.91–1.02
**Consultation with family physician**								
Native	1.00		1.00		1.00	–	1.00	
Immigrant	0.94	0.87–1.00	0.99	0.92–1.06	1.07	1.00–1.14	1.02	0.96–1.09
**Consultation with public specialist physician**								
Native	1.00		1.00		1.00	–	1.00	
Immigrant	1.02	0.90–1.16	1.06	0.94–1.21	0.97	0.86–1.09	0.92	0.82–1.04
**Consultation with private specialist**								
Native	1.00		1.00		1.00	–	1.00	
Immigrant	0.35	0.25–0.49	0.38	0.27–0.53	0.43	0.30–0.60	0.51	0.36–0.71

^a^The percentage ratio has been estimated with several regression models for each of the dependent variables that appear in the table: any consultations, consultation with family physician, consultation with public specialist physician, and consultation with private specialist. In the model 1 the adjustment variables were sex and age. In the model 2 the adjustment variables were sex, educational level, household income, household size, self-perception health and any long-term health problem

## Discussion

### Main findings of this study

In general, the percentage of people who made any medical consultation increased in 2014 with respect to 2009, in both the native and immigrant populations. Those results were due to consultations with family physicians and with public specialist physicians, since no significant differences were observed between the first and second period in the frequency of consultations with a private specialist.

In both 2009 and 2014, after adjustment for the demographic and socioeconomic variables and for the indicators of need for care, no significant differences were observed between the immigrant and native populations in the frequency of consultation with any type of physician. No were significant differences between the two populations seen in the frequency of consultation with a family physician or a public specialist physician. Only consultation with a private specialist was found to be lower in the immigrant than in the native population in both periods.

### What is already known on this topic

A previous study using data from the National Health Surveys of 2006–07 and 2011–12, which aimed to evaluate the impact of the 2008 economic crisis on the use of healthcare services in Spain, observed that the use of healthcare services was no worse in the second than in the first period in either the native population or the immigrant population [7]. The authors of the study noted that, given that the data corresponded to the period 2001–12, their investigation did not permit evaluation of the impact of the measure implemented by the Spanish government in 2012, which restricted the use of health services in undocumented immigrants. Various authors have pointed out the need to analyze the possible impact that this measure could have on the care of the immigrant population [15, 16]. The results of the present study, which used data on health services use in 2009 and 2014, show that restriction of universal health coverage has not reduced the frequency of use of health services in the in the entire population residing in Spain, natives and immigrants. Besides, the frequency of consultation with any type of physician was higher in 2014 than in 2009, an increase that was greater in the immigrant population.

According to some authors, the regional governments did not apply the measure implemented by the central government, and this could be reason for the findings in the immigrant population [17, 18]. Nevertheless, the native-born population knows better than immigrants how the healthcare system works, and this knowledge may help them to avoid entering the health system through the family physician. This could explain why the increased frequency of physician consultations in the native population was seen only for visits with public specialist physicians.

### What this study adds

There are probably various reasons for the increased frequency of physician consultation. One possible explanation could be an increase in the frequency of health problems. The findings of this investigation show that the percentage of subjects with negative self-reported health and of those with some long-term disease was higher in 2014 than in 2009, both in the native and immigrant populations. However, this reported increase by respondents is implausible from the biological point of view, at least as regards physical health problems. Furthermore, it is contrary to the trend seen for other health indicators such as life expectancy, which showed a continuous increase in Spain between 2009 and 2014 [19]. In contrast, some studies have shown an increased frequency of mental health problems in the years following the 2008 crisis [20, 21]. The estimates of the European Health Surveys used in this study also reflect this increase. The percentage of persons who reported some mental health problem in 2009 was 6% in the native population and 2% in the immigrant population, while the figures for 2014 were 7 and 4%, respectively [11]. However, given that the increase was small, it is improbable that such an increase could explain the higher frequency of physician consultations observed.

Most studies on health services use in Spain have found a greater frequency of physician consultation in the native than in the immigrant population [22]. Our findings are similar, both for 2009 and 2014. However, after adjusting for the different sociodemographic and need-for-care variables, no statistically significant differences were seen in the frequency of physician consultations between the immigrant and native populations, except for consultations with private specialists. This result suggests that the frequency of physician consultations may have increased for reasons other than the presence of health problems in the two study populations. For example, a large part of the reported increase in the frequency of both health problems and physician consultations in both populations could be a reflection of an increase in other social needs. Nor should we rule out a change in physicians’ clinical practice as responsible for this increase, especially in the case of the increased frequency of consultations with public specialist physicians. In fact, the information system of specialized physician care shows an increase in the number of consultations with public specialists per person and year [23].

The findings of the present study have great relevance. In principle, one might think that austerity policies and restrictions of access to the health system necessarily lead to a lower frequency of use of the health system. Such a thing does not have to happen, as evidenced in our study. However, from an ethical and political point of view, the achievement and maintenance of social objectives such as the human right to health care must be evaluated before the concrete results that derive from the application of those rights. Therefore, this result cannot hide that the measure adopted by the Spanish Government meant a restriction on a specific human right.

### Limitations of the study

The European Health Surveys used in this study allowed us to identify the pattern in health services use by the immigrant population in Spain before and after the measure implemented by the Spanish Government in 2012. The large amount of information offered by these surveys made it possible to control in the analyses for different variables related to the use of services. It is possible that the people most affected by this measure remained outside the sampling frame of these surveys. However, the same findings are observed in the analyses performed with data from clinical information systems in primary care. Specifically, the crude analysis shows that the frequency of consultations with primary care physicians in the immigrant population is lower than that of the native-born population [24].

In the analyzed databases it was not possible to identify the immigrants affected by the restriction on the use of health services, so the analysis we included the entire immigrant population. It is unlikely that the significant increase observed in the frequency of consultations was due exclusively to immigrants who maintained their right to health care. Perhaps, due to the fact that the regional governments did not apply the measure implemented by the central government, all immigrants contributed to this increase.

About a quarter of the selected subjects did not respond to the survey. However, there was no difference in the response rate between native and immigrant populations. On the other hand, the measures of self-perceived health problems used in health surveys may not reflect the burden of disease in the immigrant and native populations in the same way. However, data from clinical information systems in primary care show similar results: lower frequency of health problems in the immigrant than in the native population [24]. Likewise, it is possible that the measure implemented by the government in 2012 particularly affected the economically active population aged 16 to 64, whereas the present study included those aged 16 to 74 in order to increase the number of subjects analysed. Nonetheless, we performed the analyses with the sample of subjects aged 16–64 years, and the point estimates were similar.

The migrant population may have changed substantiallybetween 2009 and 2014 and this to some extent could have biasedthe results. However, such a thing did not happen if its place of origin is evaluated. The percentage of foreign population in Spain from Central and South America, Africa and Asia was, respectively, 37.7, 16.5 and 5.0% in 2009 and 38.5, 17.4 and 6.1% in 2014 [25]. However, in 2014 the proportion of the immigrant population with a low level of education was higher than in 2009. Given that subjects with a lower level of education have a higher frequency of health problems and therefore a higher frequency of use of health services, it cannot be ruled out that the increase in the frequency of doctor visits in the immigrant population may be due to this fact.

Finally, our study evaluates with 2014 data the possible effect of a measure implemented in 2012. It will be of great value to check if the pattern in the frequency of consultations to the doctor has changed, based on the information provided by the new European Health Survey in Spain, whose data has been collected throughout 2019. Mainly, because in 2018 the new Central Government repealed the measure that restricted access to the health system to undocumented immigrants.

## Conclusions

In summary, the restriction of universal health coverage in Spain did not reduce the frequency of physician consultations between 2009 and 2014, given that physician consultations increased in both the native and immigrant populations. Such a finding reflects that the impact of some political measures may be different from the theoretically expected impact, while showing that the results found should not distract attention from the ethical meaning of certain measures that restrict human rights.

## Data Availability

The databases used can be obtained from the websites of Ministry of Health and the National Statistics Institute from Spain.
